# Case Report: A Homozygous Mutation (p.Y62X) of *Phospholipase D3* May Lead to a New Leukoencephalopathy Syndrome

**DOI:** 10.3389/fnagi.2021.671296

**Published:** 2021-06-29

**Authors:** Yi-Hui Liu, Hai-Feng Zhang, Jie-Yuan Jin, Yan-Qiu Wei, Chen-Yu Wang, Liang-Liang Fan, Lv Liu

**Affiliations:** ^1^Department of Respiratory Medicine, Diagnosis and Treatment Center of Respiratory Disease, The Second Xiangya Hospital of Central South University, Changsha, China; ^2^Department of Neurology, Affiliated Hospital of Yangzhou University, Yangzhou, China; ^3^Department of Cell Biology, The School of Life Sciences, Central South University, Changsha, China; ^4^Hunan Key Laboratory of Animal Models for Human Disease, School of Life Sciences, Central South University, Changsha, China

**Keywords:** leukoencephalopathy, white matter lesions, hearing and vision loss, PLD3, Homozygous mutation, chronic kidney disease

## Abstract

Leukodystrophies are a heterogeneous group of inherited disorders with highly variable clinical manifestations and pathogenetic backgrounds. At present, variants in more than 20 genes have been described and may be responsible for different types of leukodystrophies. Members of the phospholipase D family of enzymes catalyze the hydrolysis of membrane phospholipids. Meanwhile, phospholipase D3 (PLD3) has also been found to exhibit single stranded DNA (ssDNA) acid 5′ exonuclease activity. Variants in *phospholipase D3* (*PLD3*) may increase the risk of Alzheimer's disease and spinocerebellar ataxia, but this hypothesis has not been fully confirmed. In this study, we identified a novel homozygous mutation (NM_012268.3: c.186C>G/ p.Y62X) of *PLD3* in a consanguineous family with white matter lesions, hearing and vision loss, and kidney disease by whole exome sequencing. Real-time PCR revealed that the novel mutation may lead to non-sense-mediated messenger RNA (mRNA) decay. This may be the first case report on the homozygous mutation of PLD3 in patients worldwide. Our studies indicated that homozygous mutation of *PLD3* may result in a novel leukoencephalopathy syndrome with white matter lesions, hearing and vision loss, and kidney disease.

## Introduction

Leukoencephalopathy (LE) is a structural alteration of the cerebral white matter in which myelin suffers the most damage (Kohler et al., 2018). Leukodystrophies can be broadly subdivided into hypomyelinating leukodystrophies, which are characterized by primary deficits in myelin development, and demyelinating leukodystrophies, where myelin develops normally but subsequently undergoes progressive disruption (Vanderver et al., 2015). At present, ~20 distinct disorders are defined as adulthood leukodystrophies (Tillema and Renaud, 2012; Kohler et al., 2018), such as Pelizaeus–Merzbacher disease, adult polyglucosan body disease, and X-linked adrenoleukodystrophy.

The human *PLD3* gene, which encodes a single-pass type II membrane protein with two phospholipase D (PLD) phosphodiesterase domains, is located on chromosome 19q13.2 and consists of 13 exons spanning 32 kb. As a member of the phospholipase D family of enzymes that catalyze the hydrolysis of membrane phospholipids, PLD3 has been proven to be involved in the processing of amyloid-beta precursor protein (Fazzari et al., 2017). Recently, two proteins from the PLD family, namely, phospholipase D3 (PLD3) and phospholipase D4 (PLD4), were found to exhibit single stranded DNA (ssDNA) acid 5′ exonuclease activity (Gavin et al., 2018; Cappel et al., 2020). Previous studies revealed that a heterozygous mutation of *PLD3* might increase the risk of Alzheimer's disease and spinocerebellar ataxia (Wang et al., 2015; Nibbeling et al., 2017). However, the effect of homozygous mutations in PLD3 is still not clear.

In this study, we identified a novel homozygous mutation (NM_012268.3: c.186C>G/ p.Y62X) of *PLD3* by whole exome sequencing in a patient from a consanguineous family with white matter lesions, hearing and vision loss, and kidney disease.

## Case Presentation

### Ethics Approval

This study was carried out in accordance with the guidelines of the institutional ethics committee of the Affiliated Hospital of Yangzhou University in China. All subjects gave written informed consent in accordance with the Declaration of Helsinki.

**Figure 1:**
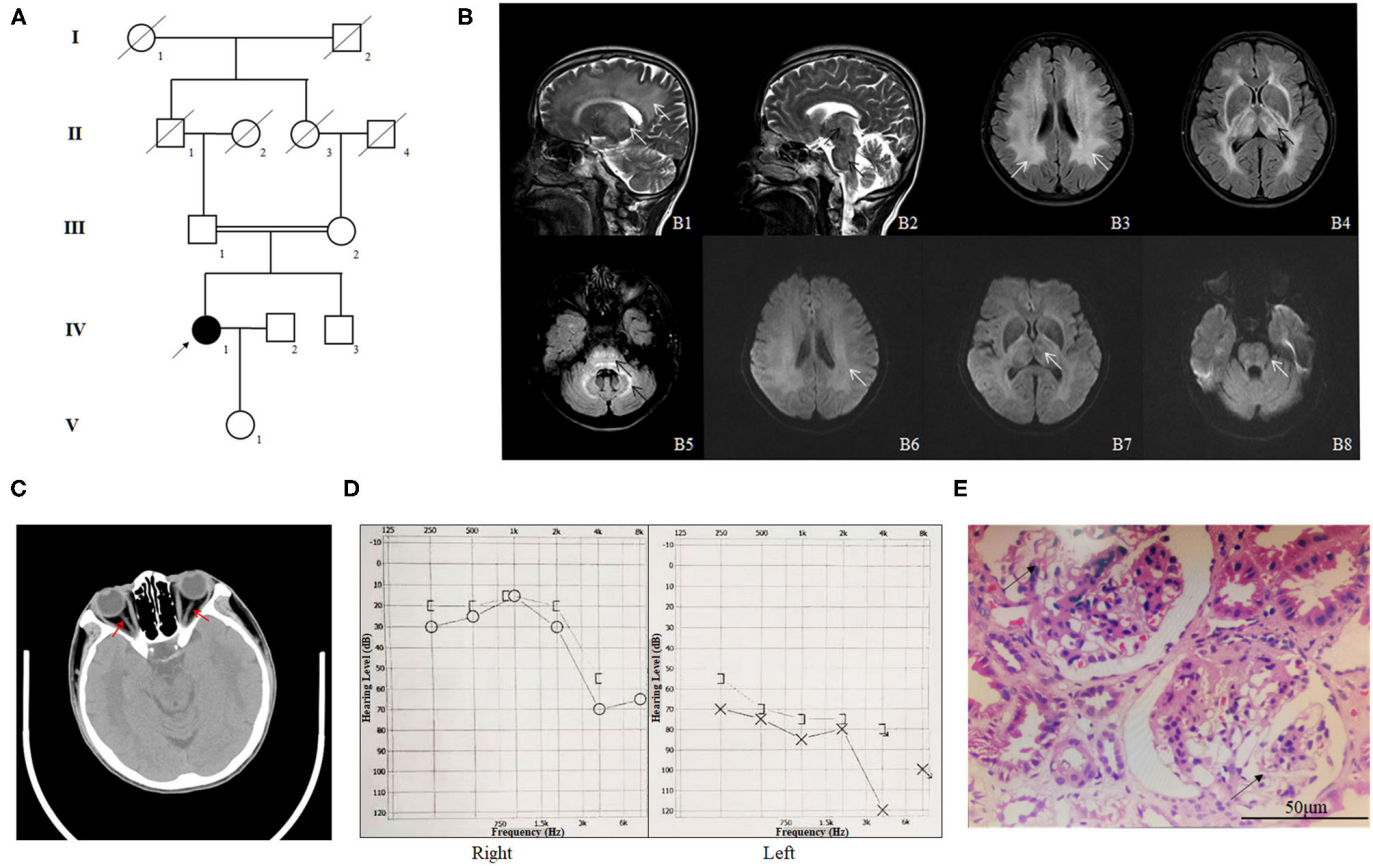
The clinical profile of the family. **(A)** Pedigree of the phospholipase D3 (PLD3)-deficient family. The pedigree chart shows five generations of the family. Roman numerals refer to generations. Circles refer to female subjects. Squares refer to male subjects. Solid symbols refer to affected subjects. Crossed-out symbols refer to deceased subjects. The arrow indicates the proband. **(B)** The MR images of the proband. T2 image shows high intensity of white matter and thalamus (**B1**, white arrows), high intensity of brain stem and thalamus (**B2**, black arrows), high intensity of bilateral white matter (**B3**, white arrows), high intensity of bilateral thalamus (**B4**, black arrows), and high intensity of brain stem and bilateral cerebellum (**B5**, black arrows). **B6–8** are the diffusion weighted image (DWI) corresponding to **B3–5** and shows slightly higher intensity. **(C)** The CT image of the proband indicating tapering of bilateral optic nerves (red arrows). **(D)** Pure-tone audiometry (PTA) results of the proband. **(E)** HE staining of the renal biopsy specimen of the proband (IV-1); the arrows indicate glomerulosclerosis.

We enrolled a consanguineous family from the Han-Chinese population (Figure 1A). The proband (IV-1), a 57-year-old woman, was admitted to our hospital due to sudden onset of lightheadedness and vertigo accompanied by nausea and vomiting. Magnetic resonance imaging (MRI) testing detected brain lesions in the proband. T2 image showed high intensity of white matter and thalamus, high intensity of brain stem and thalamus, high intensity of bilateral white matter, high intensity of bilateral thalamus, and high intensity of brain stem and bilateral cerebellum (Figure 1B). The Mini-Mental State Examination (MMSE) suggested a normal cognitive state (score, 27). Except for several episodes of lightheadedness and vertigo, the patient did not present with other motor or cognitive impairment during the hospital stay. A medical history investigation found that the proband suffered from sudden hearing and vision loss. The computed tomography (CT) image of the proband indicated tapering of bilateral optic nerves (Figure 1C). Eye examination revealed normal eye movement but a significant low vision (left, 0.3; right, 0.1). Pure-tone audiometry (PTA) showed severe hearing loss (Figure 1D). In addition, the patient also suffered from chronic kidney disease (CKD) and was diagnosed with focal segmental glomerulosclerosis by renal biopsy in another hospital 10 years ago (Figure 1E). The body mass index of the proband was 21.7, and the blood pressure was 121/77 mmHg. Blood lipid and glucose levels, as well as cerebrovascular CT of the proband, did not display any abnormalities. Upon further interviews on the family history, we found that her parents married consanguineously. The parents (III-1 and III-2) and her brother (IV-3) presented with normal vision, hearing, and kidney function. Brain MRI of the proband's parents and her brother also showed no obvious lesions (Supplementary Figure 1).

We then performed whole exome sequencing in an effort to identify the genetic lesions responsible for the disease phenotype of the proband. The central part of the whole exome sequencing was provided by the Novogene Bioinformatics Institute (Beijing, China). The exomes were captured using Agilent SureSelect Human All Exon V6 kits (Agilent Technologies, Sta Clara, CA, USA), and high-throughput sequencing was performed using Illumina HiSeq X-10 (Illumina, San Diego, CA, USA). The necessary bioinformatics analyses, including reads, mapping, variant detection, filtering, and annotation, were also carried out by Novogene Bioinformatics Institute as previously described (Fan et al., 2019).

The strategies of data filtering are as follows (Wang et al., 2020): (a) non-synonymous single-nucleotide polymorphisms (SNPs) or frameshift-causing INDELs with an alternative allele frequency >0.05 in the NHLBI Exome Sequencing Project Exome Variant Server (ESP6500), dbSNP152 (http://www.ncbi.nlm.nih.gov/projects/SNP/index.html), the 1000 Genomes project (http://www.1000genomes.org/), the ExAC database (http://exac.broadinstitute.org), or in-house exome databases of Novogene (2500 exomes) were excluded; (b) the filtered single nucleotide variants (SNVs) and INDELs, predicted to be damaging by SIFT (http://sift.jcvi.org/), Polyphen2 (http://genetics.bwh.harvard.edu/pph2/), and MutationTaster (http://www.mutationtaster.org/) were remained; (c) all the homozygous mutations were retained; and (d) cosegregation analysis was conducted in the family.

**Figure 2:**
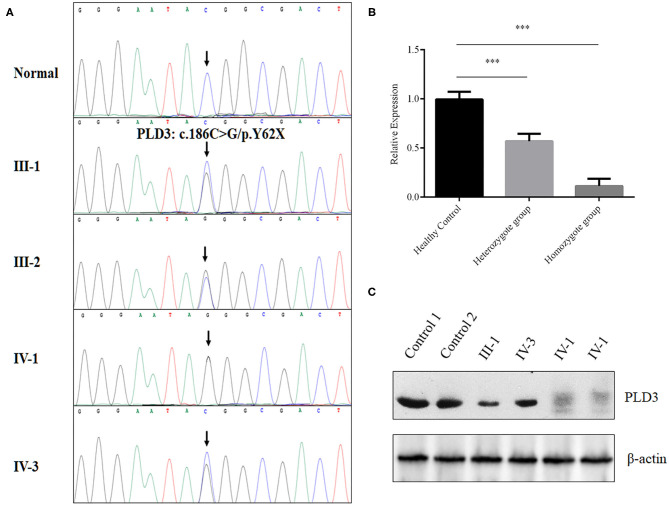
Genetic analysis of the family. **(A)** Sanger sequencing analysis of the *PLD3* gene in the patients, their family members, and the controls. **(B)** Real-time PCR determined the messenger RNA (mRNA) levels of phospholipase D3 (PLD3) in white blood cells from healthy controls (five samples), heterozygotes (III-1, III-2, and IV-3), and a homozygote (IV-1, repeated three times). ***p < 0.01. **(C)** Western blot analysis of the expression of PLD3 in white blood cells from healthy controls (two samples), the heterozygote group (III-1 and IV-3), and the homozygote group (IV-1, repeated two times).

After data filtering and American College of Medical Genetics and Genomics (ACMG) guideline assessment (Richards et al., 2015), only a novel homozygous mutation (NM_012268.3: c.186C>G/ p.Y62X) of *PLD3* identified in the proband met the likely pathogenic criteria (Supplementary Table 1). The novel non-sense mutation, resulting in a premature stop codon in exon 5 of the *PLD3* gene, was validated in the proband in a homozygous form and existed in the proband's parents and her brother in a heterozygous form (Figure 2A). We then decided to determine whether the novel variant of *PLD3* is sensitive to non-sense-mediated messenger RNA (mRNA) decay. According to the GTEx database, PLD3 is expressed in white blood cells. Hence, we isolated the total RNA from peripheral white blood cells derived from three groups or donors (five healthy controls, three heterozygote carriers, and one homozygote patient). After synthesizing complementary DNA (cDNA), real-time PCR found that the mRNA level of PLD3 in heterozygous carriers (III-1, III-2, and IV-3) was decreased by ~43% compared with that in healthy controls (five healthy people without PLD3 mutations), and the expression of PLD3 in homozygous patients (IV-1, repeated three times) was reduced by ~90% compared to that in healthy controls (Figure 2B), which indicated that the non-sense mutation may have led to non-sense-mediated mRNA decay. Concurrently, we isolated the total proteins from white blood cells obtained from two healthy people, two heterozygote carriers (III-1 and IV-3), and the proband (IV-1). Western blot analysis found that the expression of PLD3 in heterozygote group was decreased dramatically compared to healthy controls, and PLD3 was almost absent in the proband (IV-1) (Figure 2C).

## Discussion

In this study, a novel homozygous mutation (NM_012268.3: c.186C>G/ p.Y62X) of *PLD3* was identified in a consanguineous family with leukoencephalopathy syndrome by whole exome sequencing. Furthermore, real-time PCR and Western blot assays confirmed that the homozygous mutation might have led to non-sense-mediated mRNA decay and resulted in loss of function in PLD3. PLD3 is highly expressed in neurons and may regulate early neuronal development in the central nervous system (Wang et al., 2015). Meanwhile, studies found that the function of PLD3 was to catalyze the hydrolysis of membrane phospholipids. In leukoencephalopathy, neurons are damaged, and myelin is also disrupted (Kohler et al., 2018). Myelin, a phospholipids, might also be regulated by PLD3. Recently, PLD3 was found to exhibit ssDNA acid 5′ exonuclease activity (Gavin et al., 2018; Cappel et al., 2020), which was also associated with brain diseases such as Alzheimer's disease and spinocerebellar ataxia (SCA) (Gavin et al., 2018). Hence, in our case, the pathological changes in the patient who presented with high intensity white matter may have resulted from PLD3 deficiency.

Mutations in *PLD3* may also lead to endoplasmic reticulum (ER) stress and reduced phospholipase activity (Nibbeling et al., 2017). Further studies revealed that *PLD3* mutation can impair O-glycosylation at pT271 in PLD3, which is essential for normalizing antioxidative phospholipid levels and protecting the brain (Demirev et al., 2019). In addition, variants in *PLD3* can reduce PLD3 activity and affect amyloid-β levels in a cellular model of Alzheimer's disease, possibly via the autophagy-dependent mTOR signaling pathway (Tan et al., 2019). Recently, PLD3 has also been found to play a crucial role in regulating inflammatory cytokine responses (Gavin et al., 2018). Macrophages from PLD3-deficient mice had exaggerated TLR9 responses (Gavin et al., 2018). Here, in our study, the patient who carried a homozygous mutation of *PLD3* presented with white matter lesions, hearing and vision loss, and focal segmental glomerulosclerosis. We speculated that the homozygous mutation (NM_012268.3: c.186C>G/ p.Y62X) of *PLD3* may lead to PLD3 deficiency, which may induce ER stress and reduce phospholipase and exonuclease activities in neurons, as well as loss of O-glycosylation at pT271 of PLD3, ultimately damaging neuros in the central nervous system and optic and vestibulocochlear nerves. Simultaneously, PLD3 deficiency may also induce inflammatory cytokine responses in the kidney. Hence, the proband presented with phenotypes not only in the nervous system but also in the kidney. Our studies indicated that homozygous mutation of *PLD3* may result in a novel leukoencephalopathy syndrome including white matter lesions, hearing and vision loss, and kidney disease.

Previous genetic studies revealed that variants in *PLD3* may increase the risk for late-onset Alzheimer's disease (van der Lee et al., 2015; Tan et al., 2018). However, studies in Belgium found that rare variants in *PLD3* do not raise the risk for early-onset Alzheimer's disease (Cacace et al., 2015). Subsequently, Nibbeling et al. identified novel genes (*FAT2, PLD3, KIF26B, EP300*, and *FAT1*) in autosomal dominant SCA patients by whole exome sequencing. Functional studies revealed that PLD3 is located in the ER and that the missense mutation p. Leu308Pro of *PLD3* may lead to loss of function, which can induce ER stress and reduce phospholipase activity in COS-7 cells (Nibbeling et al., 2017). However, Gonzalez et al. discovered that PLD3 was located in lysosomes but not in the ER and acted as a 5′ exonuclease in HeLa cells. In addition, they also found that loss of PLD3 did not disrupt lipid catabolism and that *PLD3* knockout mice did not present cerebellar ataxia phenotypes, which challenged the interpretation of *PLD3* mutations as the causative SCA46 gene (Gonzalez et al., 2018). Hence, the identification of additional patients carrying *PLD3* mutations will further strengthen the role of PLD3 in brain disease.

In our study, four heterozygous mutation carriers (III-1, III-2, IV-3, and V-1) showed normal physical features, which indicated that heterozygous non-sense mutation of *PLD3* might not be the responsible genetic lesion of SCA and Alzheimer's disease. However, homozygous non-sense mutation of PLD3 can lead to white matter lesions, which may develop into Alzheimer's disease in the future. Certainly, we cannot exclude the genetic heterogeneity and incomplete appearance of PLD3. In fact, we enrolled almost 181 patients with white matter lesions in the past 5 years, but we only detected one homozygous mutation of PLD3 in this family. This may be the first case report on a homozygous mutation of PLD3 in patients with leukoencephalopathy syndrome. Our study may reveal a relationship between leukoencephalopathy syndrome and PLD3 homozygous mutation in patients.

In summary, we identified a novel homozygous mutation (NM_012268.3: c.186C>G/ p.Y62X) of *PLD3* in a consanguineous family with white matter lesions, hearing and vision loss, and kidney disease. This may be the first case report on a homozygous mutation of PLD3 in patients worldwide. Our study also provided new insights into function of PLD3 in human diseases.

## Data Availability Statement

The datasets presented in this study can be found in online repositories. The names of the repository/repositories and accession number(s) can be found at: https://ncbi.nlm.nih.gov/, PRJNA723675.

## Ethics Statement

The studies involving human participants were reviewed and approved by Affiliated Hospital of Yangzhou University in China. The patients/participants provided their written informed consent to participate in this study. Written informed consent was obtained from the individual(s) for the publication of any potentially identifiable images or data included in this article.

## Author Contributions

Y-HL and H-FZ enrolled the samples and performed the Sanger sequencing. J-YJ performed the real-time PCR and Western blot. Y-QW and C-YW enrolled the clinical data. L-LF and LL revised the manuscript and support the project. Y-HL, H-FZ, and J-YJ wrote the draft. All authors contributed to the article and approved the submitted version.

## Conflict of Interest

The authors declare that the research was conducted in the absence of any commercial or financial relationships that could be construed as a potential conflict of interest.
