# Supplementary material for: Case Report: A Homozygous Mutation (p.Y62X) of Phospholipase D3 May Lead to a New Leukoencephalopathy Syndrome
Source: Front Aging Neurosci. 2021 Jun 29;13:671296. doi: 10.3389/fnagi.2021.671296 (PMC8276716; doi:10.3389/fnagi.2021.671296)
Supplement: Supplementary file 1 [file Data_Sheet_1.docx]

Table S1. The genetic analysis of the proband after data filtering

| **Gene** | **Position** | **Transcript** | **hgvs.c** | **hgvs.p** | **Genotype** | **ACMGLevel** | **ACMGEvidence** |
| --- | --- | --- | --- | --- | --- | --- | --- |
| YY1AP1 | chr1:155658035-155658035 | NM_001198903.1 | c.221T>A | p.L74* | het | Likely pathogenic | pvs1 pm2 |
| FOXRED1 | chr11:126146018-126146018 | NM_017547.3 | c.875G>T | p.G292V | het | Likely pathogenic | pm1 pm2 pm5 pp3 |
| SLC12A3 | chr16:56904142-56904142 | NM_000339.2 | c.736C>A | p.L246I | het | Likely pathogenic | pm1 pm2 pm5 pp3 |
| PLD3 | chr19:40872763-40872763 | NM_012268.3 | c.186C>G | p.Y62* | hom | pathogenic | pvs1 pm2 pm3 |
| ATR | chr3:142218530-142218530 | NM_001184.3 | c.5319C>G | p.Y1773* | het | Pathogenic | pvs1 pm1 pm2 pp3 |
| ADGRV1 | chr5:89954046-89954046 | NM_032119.3 | c.4703G>A | p.S1568N | het | Likely pathogenic | pm1 pm2 ps1 |
| CFTR | chr7:117175372-117175372 | NM_000492.3 | c.650A>G | p.E217G | het | Likely pathogenic | pm1 ps1 pp3 |
| TMEM67 | chr8:94776138-94776138 | NM_153704.5 | c.475T>C | p.S159P | het | Likely pathogenic | pm1 pm2 ps1 |
| ASS1 | chr9:133355186-133355186 | NM_000050.4 | c.772G>A | p.A258T | het | Likely pathogenic | pm1 pm2 pm5 pp3 |
| YY1AP1 | chr1:155658033-155658033 | NM_001198903.1 | c.222_223insAA | p.A75Kfs*65 | het | Likely pathogenic | pvs1 pm2 |
| MLYCD | chr16:83932809-83932809 | NM_012213.2 | c.60del | p.R21Gfs*52 | het | Likely pathogenic | pvs1 pm2 |
| CYP24A1 | chr20:52779402-52779402 | NM_000782.4 | c.845-2dup | . | het | Pathogenic | pvs1 pm2 pp3 |
| NPC1L1 | chr7:44578722-44578723 | NM_013389.2 | c.1273_1274del | p.L425Afs*120 | het | Likely pathogenic | pvs1 pm2 |
| ASPH | chr8:62550924-62550924 | NM_004318.3 | c.791-4_791-3dup | . | het | Pathogenic | pvs1 pm3 pp3 |


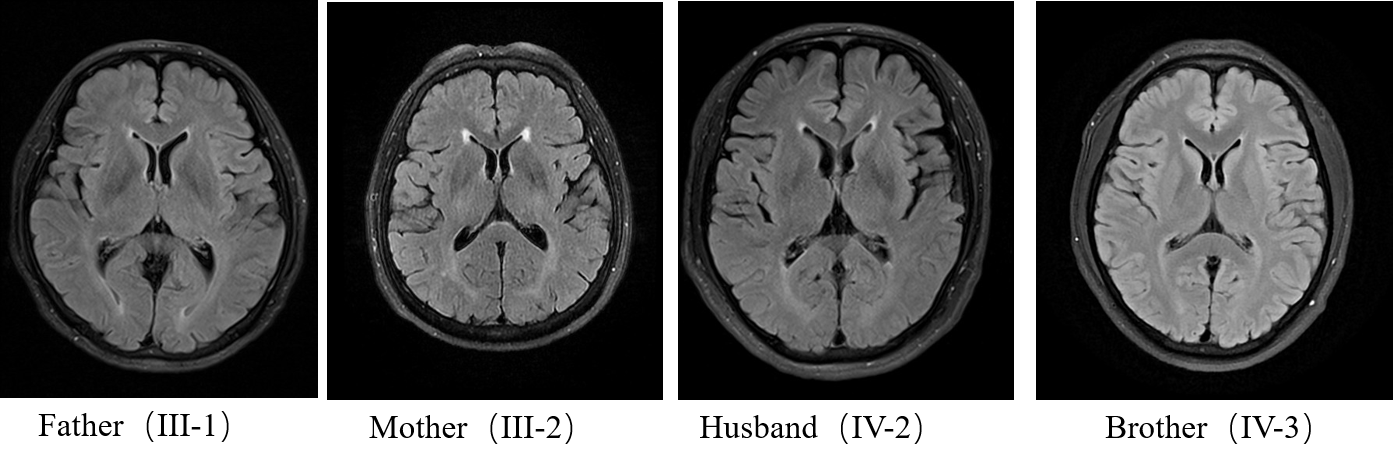


Figure S1. The MRI of other family members.The T2 FLAIR image of other family members didn’t show high intensity of white and gray matter.
